# Psychrotrophic Antarctic marine bacteria as potential reservoirs for novel antimicrobial genes

**DOI:** 10.1093/femsmc/xtaf004

**Published:** 2025-04-15

**Authors:** Kudzai Hwengwere, Grant G January, Kerry L Howell, Lloyd S Peck, Mathew Upton, Melody S Clark

**Affiliations:** British Antarctic Survey, Natural Environment Research Council, Cambridge CB3 0ET, United Kingdom; School of Biological and Marine Sciences, University of Plymouth, Plymouth, PL4 8AA, United Kingdom; School of Biomedical Sciences, Derriford Research Facility, University of Plymouth, Plymouth, PL6 8BU, United Kingdom; School of Biological and Marine Sciences, University of Plymouth, Plymouth, PL4 8AA, United Kingdom; Plymouth Marine Laboratory, Plymouth, PL1 3DH, United Kingdom; British Antarctic Survey, Natural Environment Research Council, Cambridge CB3 0ET, United Kingdom; School of Biomedical Sciences, Derriford Research Facility, University of Plymouth, Plymouth, PL6 8BU, United Kingdom; British Antarctic Survey, Natural Environment Research Council, Cambridge CB3 0ET, United Kingdom

**Keywords:** OSMAC, thermal tolerance, biosynthetic gene cluster, salinity, invertebrate, microbial

## Abstract

Antarctica is a very cold, isolated continent surrounded by frozen seas, yet these extreme environmental conditions have not restricted life and diversity in the sea. The marine environment is seasonally highly productive and harbours diverse and abundant communities of organisms, with many endemic species occurring nowhere else in the world. Such communities and their associated microbiomes are increasingly recognized as an unexplored source of novel antimicrobial products. Hence, the major aim of this study was to examine the antimicrobial potential of bacteria cultured from eight Antarctic marine invertebrate species, while gathering data on Antarctic microbial thermal and salinity tolerances. All cultured bacterial species (*n* = 34) were related to known psychrotrophs, with thermal tolerances that far exceeded those of their invertebrate hosts. Of note, two strains of *Psychrobacter* and *Pseudomonas* produced antagonistic activity towards epidemic methicillin-resistant *Staphylococcus aureus, Micrococcus luteus*, and *Candida albicans* in preliminary simultaneous antagonism screens. Draft whole genome sequence analysis revealed the presence of 13 biosynthetic gene clusters; including those with potential to produce betalactones, post-translationally modified peptide products, and arylpropynes. These results emphasize the need for more extensive and systematic surveys to identify novel biomolecules from Antarctic marine bacteria that may be exploited for societal gain.

## Teaser

Bacteria taken from Antarctic marine creatures live in a perpetually cold environment but can grow at moderate temperatures and produce novel chemicals for use against human disease.

## Introduction

Early scientific literature underestimated Antarctic biodiversity, predicting that the extreme environmental conditions would deter the persistence of a rich and biodiverse ecosystem (Janosik and Halanych 2010). While true for groups such as gastropods and bivalves, advances in biodiversity survey and monitoring have shown the highly biodiverse nature of Antarctic marine fauna, notwithstanding many endemic species (Chown et al. 2015, Peck 2018). More recently, the importance of cold-adapted Antarctic microbes is attracting attention. Microbes account for the largest proportion of biomass on the continent and are a potential untapped source of chemically distinct natural products of biotechnological value (Bruno et al. 2019, Ortiz et al. 2021). For example, metagenomic analysis of uncultured bacteria from Antarctic terrestrial and marine environments has revealed an abundance of biosynthetic gene clusters (BGCs) with high genetic diversity and predicted antimicrobial capacity (Waschulin et al. 2022, Cho and Lee 2024, Medeiros et al. 2024, Medeiros et al. 2025). In addition, cultured Antarctic bacteria have demonstrated inhibition of pathogenic bacteria, and notable examples include an Antarctic strain of *Serratia myotis*, which displayed antibiotic activity against seven pathogenic bacteria (Xiao et al. 2023), and the Antarctic cyanobacterium Nostoc CCC537 which produced an antibacterial lead molecule active against two Gram-positive pathogens and seven Gram-negative bacteria, including three multidrug-resistant strains of *Escherichia coli* (Asthana et al. 2009).

Cold-adaptation strategies employed by Antarctic bacteria and their association with the production of atypical antimicrobials have been reviewed by Ramasamy et al. (2023) and Núñez-Montero and Barrientos (2018), respectively. These reviews discussed several strategies, such as altering cell membrane structure, synthesizing antifreeze proteins to help survive sub-zero temperatures, and modifying metabolism and pigment production for antioxidation, photoprotection, and antimicrobial effects. Of note, antimicrobial production can mediate microbial antagonistic interactions and provide producers with a competitive advantage in populated niches (Long and Azam 2001). Several studies have observed considerable bacterial antagonism in polar soils (Bell et al. 2013). This adaptation is thought to be a response to the nutrient-poor terrestrial environment and the battle for resources among the endemic bacteria and fungi (Bell et al. 2013). O’Brien et al. (2004) isolated bacteria from Antarctic soils and identified that 0.29% of bacteria showed antagonistic interactions and bioactivity against food-borne pathogens using a deferred antagonistic assay (O’Brien et al. 2004). Prasad et al. (2011) identified 14% of cultured bacteria from sampled Arctic soils displayed antagonistic relationships. Despite fewer studies on antagonism in Antarctic marine environments, there have been promising reports (Lo Giudice et al. 2007). Lo Giudice et al. (2007) cultured Antarctic marine bacteria with antagonistic activity against a range of pathogenic bacteria, such as *E. coli, Micrococcus luteus, Bacillus subtilis*, and *Proteus mirabilis*, while Maida et al. (2015) cultured an Antarctic marine *Pseudoalteromonas* active against opportunistic pathogens associated with cystic fibrosis. Additionally, as bacteria in ecological niches with higher densities of microbes and confined space show higher levels of antagonism than free-living bacteria, Antarctic marine invertebrate microbiomes may act as vehicles of bioactive bacteria, e.g. an Antarctic Annelid with hydrolytic enzyme producing microbes (Herrera et al. 2017). Lo Giudice and Rizzo (2018) reviewed the isolation of bioactive bacteria from Antarctic marine invertebrates. Regardless of demonstrated potential, paricularly from sponge symbionts, the antimicrobial potential of bacteria from other dominant invertebrate taxa inhabiting the West Antarctic Peninsula continental shelf, such as Echinodermata, Cnidaria, and Annelida phyla, is hugely understudied (Mangano et al. 2009, González-Aravena et al. 2016, Lo Giudice et al. 2019).

Culturable bacteria represent <1% of all bacteria present in the environment, but they are an invaluable resource for the identification and functional characterization of natural products (Staley and Konopka 1985, Lo Giudice and Rizzo 2018). Moreover, culturing approaches can be streamlined to increase the efficiency of antimicrobial discovery. A prime example is where bacterial strains are subjected to varied culture conditions as a means of stimulating silent BGCs and capturing more diverse chemicals (Núñez-Montero et al. 2020), which includes the ‘One Strain-Many Compounds’ (OSMAC) approach (Bode et al. 2002). Hence, the major aims of this study were to (i) characterize and determine whether bacteria cultured from eight marine invertebrate species show antimicrobial potential, (ii) assess the utility of OSMAC for enhancing the success rates of antimicrobial screening, and (iii) genome mine productive bacterial isolates for novel antimicrobial gene properties. Temperature and salinity were chosen as OSMAC conditions on account of their capacity to modulate bacterial growth rates and stimulate or reduce their production of secondary metabolites (Ng et al. 2014, Núñez-Montero et al. 2020). Furthermore, baseline data on thermal and salinity tolerances of the cultured bacteria were collected.

## Methods

### Sampling

Eight species of common Antarctic marine invertebrates (the starfish, *Odontaster validus*; the brittle star, *Ophionotus victoriae*; the sea cucumbers, *Echinopsolus charcoti, Cucumaria georgiana*, and *Heterocucumis steineni*; the sea urchin, *Sterechinus neumayeri*; the limpet, *Nacella concinna*; and the anemone, *Urticinopsis antarctica*) were collected by SCUBA divers from South Cove, Ryder Bay, Adelaide Island, and West Antarctic Peninsula (67°34 S, 68°08 W), in March 2020, June 2020, and February 2022, at depths of 15–20 m. These eight invertebrate species were selected for sampling because they are highly abundant in the marine environment at Ryder Bay, have been the subject of many physiological studies, and significantly contribute to the functioning of Antarctic benthic ecosystems (Peck 2018).

### Culturing of bacteria

Six animals were collected in March 2020 (*O. validus; O. victoriae; H. steineni; S. neumayeri; N. concinna*, and *U. antarctica)* and two in June 2020 (*C. georgiana; Ec. charcoti*). Immediately after collection, the animals were frozen at −20°C before return to the UK. In the UK, the animals were dissected into their discrete tissue sections (body wall, gut, and tentacle, dependent on animal) to maximize bacterial recovery as bacteria found on body wall, gut, or tentacle experience very different microenvironments (Hughes et al. 2022). One gram of each tissue type was homogenized in 9 ml of phosphate buffered saline and diluted by a factor of 50 and 100. Aliquots (500 µl) of the homogenate, neat and dilutions, were spread onto three types of solid isolation media [Reasoner’s 2A (R2A), Marine Broth, and Actinomycetes media] and cultured at 4°C and 15°C for 6–8 weeks, following published protocols (Koch et al. 2019). As the bacterial population may have been modified by freezing and shipping at −20°C, all eight species of invertebrates (*O. validus, O. victoriae, H. steineni, S. neumayeri, N. concinna, U. antarctica, C. georgiana*, and *Ec. charcoti*) were additionally collected in February 2022 and harvested for bacteria immediately after collection (as described above) on site at Rothera, a UK Antarctic Research Station. The bacterial isolates recovered were then shipped via the Royal Research Ship (RRS) Ernest Shackleton on plates at 4°C to the British Antarctic Survey (BAS) laboratories in Cambridge, UK.

### 16S rRNA-based molecular identification of bacterial colonies

Morphologically distinct bacterial colonies were subjected to molecular barcoding using the 16S rRNA gene, V3–V4 region [primers 341F (CCTAYGGGRBGCASCAG) and 806R (GGACTACNNGGGTATCTAAT)] (Yu et al. 2005). These primers were selected to allow for future comparisons with 16S rRNA amplicon sequencing projects targeting the same region. Polymerase chain reaction (PCR) amplification was performed using MyTaq™ 2x Red Mix (Meridien Biosciences) according to manufacturer’s instructions and the ∼470 bp product was Sanger sequenced by Source Bioscience, UK. The resulting reverse and forward sequences were merged using Geneious Prime version 2023.2.1. NCBI BLASTn version 2.12.0+ and 2.13.0+, was run on Geneious Prime and used to search the merged sequences against the NCBI 16S ribosomal RNA database, in November 2022 and July 2023, to allow taxonomic identification of the bacterial isolates. To ensure a manageable number of bacterial strains for the OSMAC treatments and activity testing, isolates with >99.9% match in their 16S rRNA gene sequences and identical closest species based on the BLASTn results, were dereplicated. All 16S rRNA gene sequences have been submitted to GenBank and the accession numbers are provided in the data availability section.

## OSMAC approach to elicit antimicrobial activity

An OSMAC approach was taken to test the impact of lowering salinity and raising temperature to near stressful conditions, on antimicrobial production. Suitable temperature parameters to use in the OSMAC experiments were decided by a literature search of the thermal tolerances of the closest matching species of the cultured bacteria. The control temperature and salinity conditions in the OSMAC experiment were a replica of the conditions originally used to culture the bacterial isolates from the eight invertebrate hosts. A salinity of 10‰ was used based on a previous report that Antarctic bacteria isolated from ice-free marine areas showed signs of cellular stress at this salinity (Martin et al. 2009). Bacteria were initially cultured in Marine Broth at 4°C until they attained an optical density of 0.1 ml^−1^ at a wavelength of 600 nm (OD_600_). Then, 40 µl of this original culture was inoculated in 4 ml of Marine Broth under the different temperature and salinity conditions outlined below. The OSMAC experiment comprised a Latin square design, containing four treatments [full controls (4°C and 22‰); control temperature (4°C) and reduced salinity (10‰); elevated temperatures (see below for details) and control salinity (22‰) and finally combined elevated temperature and reduced salinity (10‰)]. For the elevated temperature experiments, bacteria were incubated at either 22°C, 28°C, or 35°C, depending on the maximum temperature tolerated by their closest relative. There were triplicates and media blanks with no bacterial inoculation (negative controls) in each treatment. On the seventh and fourteenth day of incubation, each sample was checked for signs of turbidity, and a 1 ml aliquot of each sample was taken. The aliquots were centrifuged at 7378 × *g* for 10 min and preserved at −20°C for antimicrobial testing using the agar-well diffusion assay. Freezing samples at −20°C was performed to facilitate simultaneous and bulk processing of antimicrobial tests. In addition, on the fourteenth day, all the liquid cultures, including the media blanks, were streaked onto R2A agar and incubated at 15°C for a minimum of 10 days, to verify the purity of the cultures with further confirmatory sequencing of the 16S rRNA gene, V3–V4 region. The incubation conditions were selected to allow faster growth and added time for bacteria or slow-growing contaminants, if present, to appear. All media blanks were contaminant-free.

### Agar well-diffusion assay

The well-diffusion assay was performed according to Lertcanawanichakul and Sawangnop (2008). A few alterations were made as described below. Testing for antimicrobial activity was performed against *Acinetobacter baumannii* (strain ATCC 19606), *Candida albicans* SC5314 (strain ATCC MYA-2876), *E. coli ST-38* (an in-house clinical UPEC isolate), Epidemic *Methicillin-resistant Staphylococcus aureus* (in-house clinical strain 17) (EMRSA), and *M. luteus* (strain NCTC 2665). All strains were sourced from the University of Plymouth, Derriford Research Facility and were chosen based on availability as well as, representation of different groups, i.e. Gram-positive, Gram-negative, and a fungal group. The test pathogens were cultured to an OD_600_ of 0.1 at 37°C. Aliquots (100 µl) of this were spread onto Mueller–Hinton agar plates. Wells of 6 mm diameter were dug into the agar. Bacterial aliquots that had been centrifuged and stored at −20°C were thawed and 50 µl was added to each well. The plates were incubated at 37°C for 24–72 h. The zone of inhibition was quantified by measuring the diameter (mm) of pathogen clearance around the wells and subtracting 6 mm. The agar well-diffusion test was also repeated with an additional 10-min centrifugation step (16 162 × *g*) of the bacterial aliquots, prior to well inoculation, to ensure the removal of bacterial cellular material from the culture media.

#### Cross-streak assay

Bacterial isolates showing antimicrobial activity were also subjected to the deferred antagonism method described by Kékessy and Piguet (1970). A single streak of the Antarctic bacteria was left to grow on Mueller–Hinton agar plates at 4°C and 28°C for 7 days. *Candida albicans*, EMRSA, and *M. luteus* were diluted to an OD_600_ ml^−1^ of 0.1 and streaked perpendicularly to the Antarctic strain bacterial streaks that had been incubated for 7 days. It was ensured that there was a gap left between the perpendicular streaks of the pathogens and the streak of the Antarctic bacteria (no physical contact) to prevent growth inhibition resulting from bacteriophage contamination. There were five replicates in each treatment. The plates were incubated at 37°C for 24 h to allow the pathogen to grow. A control plate with the pathogens alone was used as a negative control.

### Whole genome assembly

The strains (AG3 and AT9) that produced antimicrobial activity were taken for whole genome sequencing to allow identification of the BGCs in the strains and the bioactive products the strains may produce. The strains were cultured on R2A agar plates and incubated at 4°C for 10 days, before being sent to MicrobesNG for genomic DNA extraction and sequencing. Genomic DNA was extracted by lysing 5–40 µl of the cell suspension in 120 µl of TE buffer with lysozyme (MPBio, USA), metapolyzyme (Sigma–Aldrich, USA), and RNase A (ITW Reagents, Spain). The lysate was subjected to a 25 min incubation at 37°C. Following on, the lysate was incubated for a period of 5 min at 65°C together with proteinase K (VWR Chemicals, OH, USA) at a final concentration of 0.1 mg ml^−1^ and SDS (Sigma–Aldrich, MO, USA) at a final concentration of 0.5% v/v. An equal volume of SPRI beads were used to purify the DNA before resuspending it in EB buffer (10 mM Tris-HCl, pH 8.0). DNA was quantified using the Quant-iT dsDNA HS (Thermo Fisher Scientific) assay in an Eppendorf AF2200 plate reader (Eppendorf UK Ltd, UK) and diluted as appropriate. For Illumina sequencing, genomic DNA libraries were prepared using the Nextera XT Library Prep Kit (Illumina, San Diego, CA, USA) according to the manufacturer’s protocol. Slight adjustments were made, whereby the input DNA was doubled, and the PCR elongation time extended by 45 s. The genomes were sequenced on an Illumina NovaSeq 6000 using a 250 bp paired-end protocol. Reads were adapter trimmed using Trimmomatic version 0.30. SPAdes version 3.7 was used for de novo assembly and Prokka version 1.11 for contig annotation. For the hybrid approach, DNA libraries were prepared with Oxford Nanopore SQK-LSK109 kit with Native Barcoding EXP-NBD104/114 (ONT, UK) using 400–500 ng of high molecular weight DNA. Barcoded samples were pooled together into a single sequencing library and loaded in a FLO-MIN106 (R.9.4.1) or FLO-MIN111 (R10.3) flow cell in a GridION (ONT, UK). The short and long reads underwent hybrid assembly using Unicycler version 0.4.0 and contig annotation performed using Prokka version 1.11. All analyses were performed using default parameters. MicrobesNG produced the genome assemblies, performed contig annotation using Prokka and provided the assembly statistics in Table S4. The raw sequencing reads, hybrid assemblies, and Prokka annotations are available from SRA, GenBank, and Zenodo, respectively. The relevant accession numbers and digital object identifier (DOI) are provided in the data availability section.

## Taxonomic and phylogenetic analysis

The whole genome sequences were uploaded onto online taxonomic servers and compared against the bacterial reference genome database on JSpecies Web Server, to identify species match and novelty (Richter et al. 2016). The comparison was done using average nucleotide identity (ANI), based on BLAST + (ANIb) calculations. ANIb measures the extent of similarity of DNA molecules between two genomes and a value greater than 95% or 96% between two strains is regarded as a positive species match (Goris et al. 2007). These values were verified against digital DNA–DNA hybridization (dDDH) values calculated from the genome-to-genome distance calculator (GGDC) GGDC 3.0 (Meier-Kolthoff et al. 2021). The dDDH calculation is an important criterion for determining the relatedness between strains and a score of 70% or more is regarded as a species match. Phylogenetic trees of AG3 and AT9 and their closest related type strains were constructed in the Type Strain Genome Server (TYGS) (Meier-Kolthoff et al. 2021). The phylogenetic trees were generated based on whole genome sequences and inferred using FastME 2.1.4 and Genome BLAST Distance Phylogeny (GBDP) pseudo-bootstrap from 100 replications. All software above were accessed and used to generate data in this paper on the 21st of February 2024. Inkscape version 1.4 was used to make final adjustments to the phylogenetic trees.

### *In silico* genome mining for BGCs

To consider the preliminary identities of the antimicrobial compounds produced by the active bacterial strains, their *in silico* identified BGCs were analysed using Antimicrobial Secondary Metabolite Analysis Shell version.7.0 (antiSMASH) (Blin et al. 2023). The antiSMASH results were queried against the Biosynthetic Gene Cluster Family’s Database (BiG-FAM) version 1.0.0 (Kautsar et al. 2021). The Antibiotic Resistant Target Seeker version 2.0 (ARTS) programme was used to predict the BGCs with the highest likelihood of producing antibiotic compounds. ARTS uses machine learning algorithms to predict the proximity of core genes, horizontal gene transfer, duplication of genes, and the presence of resistance genes, all of which, when present, suggest that the BGC can produce an active compound (Mungan et al. 2020). Two BGCs showed positive hits for resistance genes in ARTS analysis. The sequences corresponding to the two BGCs and the full hybrid assemblies of AG3 and AT9 were examined for putative antimicrobial resistance (AMR) genes through Comprehensive Antibiotic Resistance Database (CARD) version 4.0.0 and Resistance Gene Identifier (RGI) version 6.0.3 (Alcock et al. 2023). If no positive hits were obtained using default parameters, then the search criteria were adjusted from ‘Perfect and strict hits only’ to ‘Perfect, strict, and loose hits’ for the identification of potential new AMR genes. AntiSMASH, ARTS, BiG-FAM, and CARD/RGI were accessed and used to generate data in this paper on the 21st of February 2024, 31st of July 2024, 16th of January 2025, and the 31st of March 2025, respectively. All programmes were run using default parameters, unless stated otherwise. The outputs from AntiSMASH and CARD analysis have been deposited to Zenodo and the relevant DOI is provided in the data availability section.

## Results

### Diversity of culturable bacteria from Antarctic marine invertebrates

**Figure 1.**
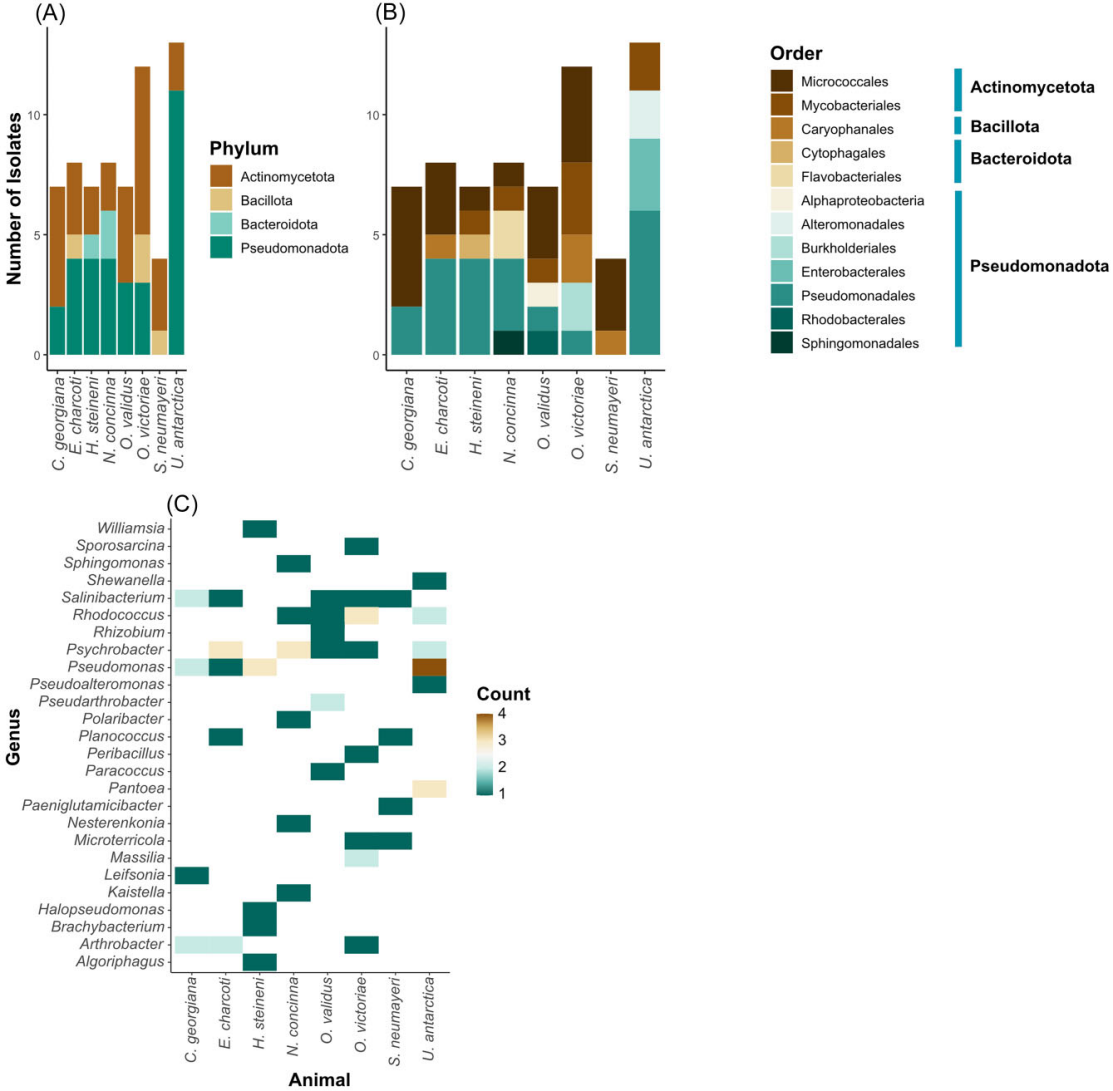
Bacteria cultured from eight Antarctic marine invertebrates and identified using molecular barcoding of the 16S rRNA gene, V3–V4 region. A total of 66 bacterial isolates were cultured from eight Antarctic invertebrates (*C. georgiana, Ec. charcoti, H. steinini, N. concinna, O. validus, O. victoriae, S. neumayeri*, and *U. antarctica*). Bacteria were cultured from two individuals of each species, one processed with freezing and one processed without freezing, and the figure represents the total of the two individuals per species. Bacteria were identified according to the 16S rRNA gene, V3–V4 region and grouped according to (A) phylum, (B) order, (C) and genera, where the count corresponds to the number of isolates. For the full listing and associated references, see Table S1.

A total of 66 bacterial isolates were identified with 16S rRNA gene sequencing of the V3–V4 region. The 66 isolates were representative of four phyla, 26 genera, and 34 closest matching species (Fig. 1). The isolates comprised members of the phyla, Actinomycetota (formerly Actinobacteria) (49%), Pseudomonadota (formerly Proteobacteria) (41%), Bacillota (formerly Firmicutes) (6%), and Bacteroidota (formerly Bacteroidetes) (4%). The diversity of bacterial taxa varied across the eight host species. Only bacteria from the Actinomycetota and Bacillota phyla were isolated from *S. neumayeri*. The animals *C. georgiana, O. validus*, and *O. victoriae* had a higher richness of Actinomycetota as compared to Pseudomonadota, whereas *H. steinini* and *Ec. charcoti* were similarly rich in Actinomycetota and Pseudomonadata, and each contained a member of the Bacteroidota and Bacillota phyla, respectively. In contrast, the mollusc, *N. concinna*, and cnidarian, *U. antarctica*, were richer in Pseudomonadota, with no representation of Bacteroidota and Bacillota. Interestingly, all the bacterial isolates belonging to the phylum Bacteroidota and the genus *Pseudomonas* were only identified in freshly sampled animals. In addition, only bacterial isolates belonging to the genera *Arthrobacter, Microterricola, Psychrobacter*, and *Salinibacterium* were isolated from both fresh and frozen samples. Isolate AT5, belonging to the genus *Pseudomonas*, was only cultured in freshly sampled animals, but was the most common, being found in five species. Of the 34 unique bacterial species, 17 (50%) were closely related to strains identified in polar regions or other cold environments. Of these 17 strains, nine were related to species that were isolated in the Antarctic, and these were almost equally distributed between the terrestrial and marine environment (five and four, respectively). Two closest relatives were identified in the Arctic. The remaining six closest relatives were from cold non-polar regions, such as a glacier in China and marine trenches in Japan and the Pacific Ocean (Table S1).

### OSMAC experiments

**Figure 2.**
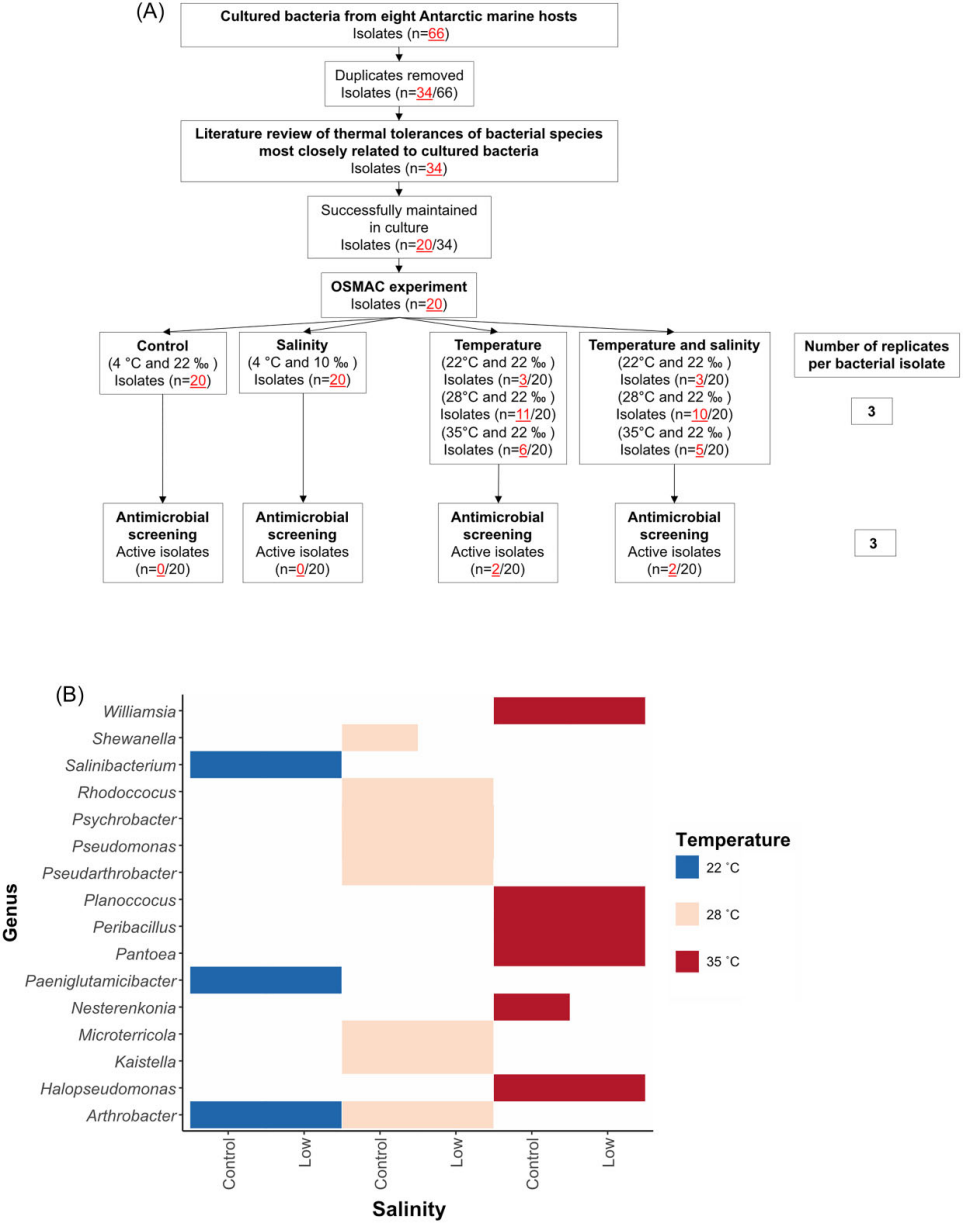
Flowchart of bacterial isolates from initial culturing to antimicrobial testing and isolates in OSMAC temperature treatments. (A) The flowchart represents the number of bacterial isolates that were initially cultured from eight invertebrate hosts (66) and how many of these were gradually filtered and used for the OSMAC experiments. After dereplication, 20 isolates were successfully grown in culture and subjected to the OSMAC experiment and tested for antimicrobial activity. The numbers of bacterial strains that grew in each OSMAC treatment are provided. (B) Upper thermal tolerances of the 20 bacterial isolates grown under OSMAC conditions and grouped according to genera. Isolates were grown under control salinity of 22‰ (Control) and low salinity of 10‰ (Low). At both salinities, isolates were incubated at either 35°C, 28°C, or 22°C. For further details into the identities of the strains and their associated thermal tolerances, see Table S2.

The 66 cultured bacteria were filtered to 34 strains through dereplication of identical bacterial species based on their 16S rRNA gene sequences (Fig. 2A). For all 34 strains a literature search was conducted to examine the thermal tolerances of their closest bacterial species (based on BLASTn search results). According to the literature searches, none of the 34 strains were ‘true’ psychrophiles, as their thermal limits all exceeded 20°C (Moyer et al. 2017). Nevertheless, six strains had reported thermal limits close to 20°C (between 22°C and 25°C) and were related to isolates from cold environments. Of these six strains, one was isolated from a glacier in China, four in the Antarctic, and one in the Arctic. The remaining 28 isolates had closest relatives that could tolerate temperatures greater than 28°C, with 17 surviving at least 35°C, and four surviving 40°C (Table S2). Hence 22°C, 28°C, and 35°C were used as the OSMAC temperature stressors.

Of the 34 bacterial strains, 20 were successfully maintained in culture, and used for the OSMAC experiments and antimicrobial testing. Nearly all 20 isolates showed visible signs of growth at the test temperature suggested by the literature survey, except for five strains at 35°C. The five strains that did not mount visible growth at 35°C were alternatively grown at 28°C and survived. In terms of salinity, all 20 isolates showed turbidity in low salinity (10‰) media when incubated at control temperatures (4°C). At test temperatures, 18 isolates showed growth in low salinity media. Conversely, two isolates, 6F13 (*Nesterenkonia* sp.) and 2T37 (*Shewanella* sp.) displayed growth at their test temperatures (35°C and 28°C, respectively) and control salinity, but not at the combination of test temperature and low salinity (Fig. 2B). No antibiotic production was observed in the 20 isolates grown in the OSMAC control conditions (4°C and 22‰). Under the OSMAC conditions, 18 isolates displayed no antimicrobial activity. However, two strains [AG3 and AT9 (*Pseudomonas* sp. and *Psychrobacter* sp., respectively)] displayed antimicrobial activity, when grown at 28°C and both 22‰ and 10‰ salinity (Table 1).

**Table 1.** Temperature tolerance, salinity tolerance, and antimicrobial activity data of 20 cultured isolates in liquid culture.

	Closest related strain according to 16S rRNA gene, V3–V4 region	Thermal tolerance		Salinity tolerance	Growth at low salinity and thermal stress	
Isolate code		Projected (°C)	Observed (°C)	Observed (‰)		Antimicrobial activity
HS1W	*Halopseudomonas* sp.	37	35	10	Yes	No
6F13	*Nesterenkonia* sp.	40	35	10	No	No
2B3	*Pantoea* sp.	42	35	10	Yes	No
5S34	*Peribacillus* sp.	40	35	10	Yes	No
PS9	*Planoccocus* sp.	37	35	10	Yes	No
AG3	*Pseudomonas* sp.	37	28	10	Yes	Yes
AT9	*Psychrobacter* sp.	36	28	10	Yes	Yes
1S15	*Williamsia* sp.	30	35	10	Yes	No
PS2.1	*Pseudarthrobacter* sp.	30	28	10	Yes	No
NG2	*Kaistella sp*.	28	28	10	Yes	No
SG4	*Microterricola* sp.	30	28	10	Yes	No
4S15	*Arthrobacter* sp.	30	28	10	Yes	No
AT5	*Pseudomonas* sp.	30	28	10	Yes	No
6F3	*Psychrobacter* sp.	30	28	10	Yes	no
6F8	*Psychrobacter* sp.	37	28	10	Yes	No
6G9	*Rhodoccocus* sp.	35	28	10	Yes	No
2T37	*Shewanella* sp.	30	28	10	No	No
SG15	*Paeniglutamicibacter* sp.	24	22	10	Yes	No
4S16	*Salinibacterium* sp.	23	22	10	Yes	No
CS7	*Arthrobacter* sp.	25	22	10	Yes	No

For full listing see Table S2.

### Antagonistic antimicrobial activity of strains AT9 and AG3

**Figure 3.**
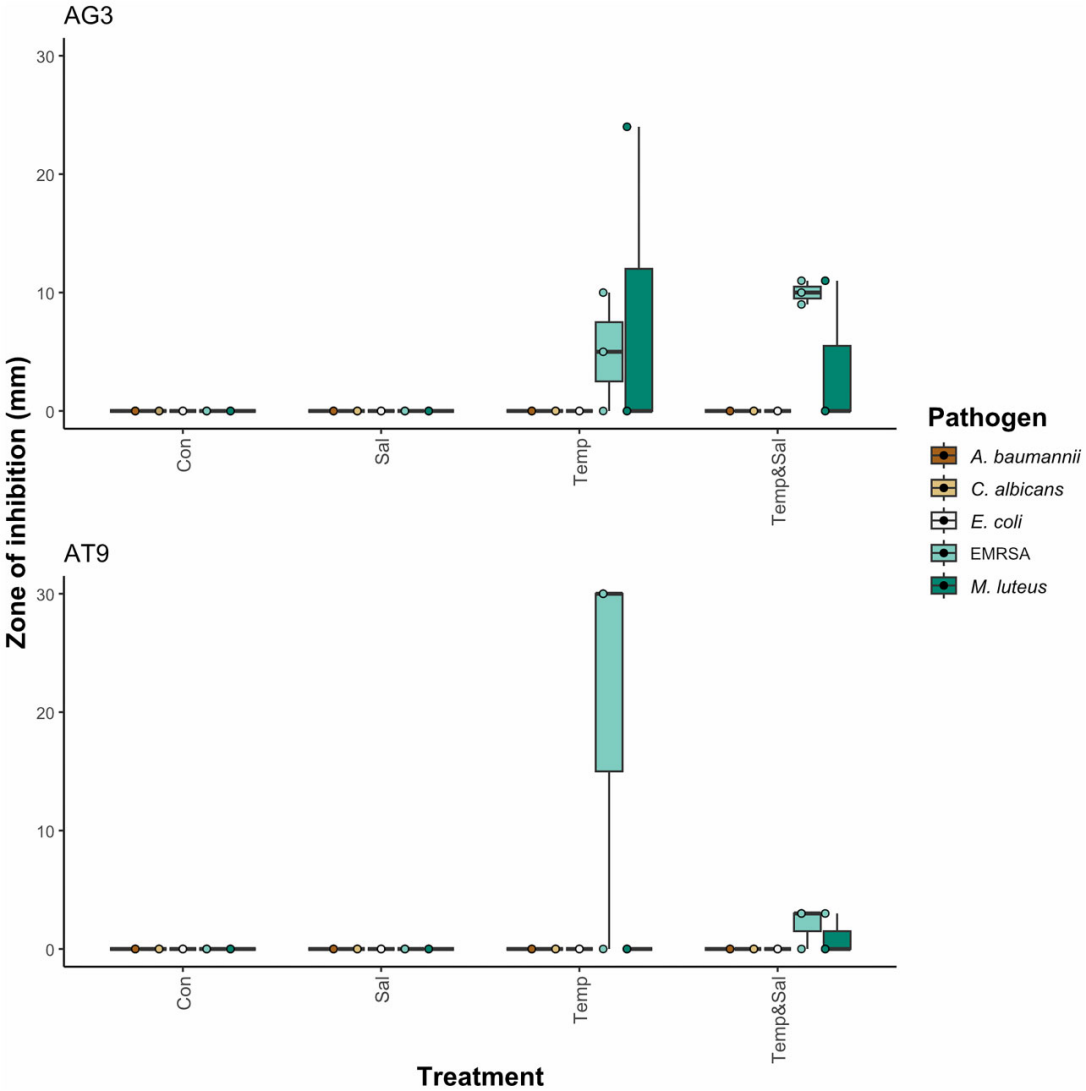
Antimicrobial activity of AG3 and AT9 in an agar well-diffusion assay. The zone of inhibition is the diameter (mm) of the area of pathogen clearance. Triplicates of each OSMAC treatment were tested against five pathogens (*A. baumannii, C. albicans, E. coli*, EMRSA, and *M. luteus*) and the conditions were as follows: control (4°C and 22‰), salinity (4°C and 10‰), temperature (22°C or 28°C or 35°C and 22‰), and temperature and salinity (22°C or 28°C or 35°C and 10‰). Abbreviations: Con, control; Sal, salinity; Temp, temperature; and Temp&Sal, temperature and salinity.

The antimicrobial activity of recovered strains AG3 and AT9 against EMRSA appeared to be antagonistic because growth inhibition was only observed around wells where AG3 and AT9 had regrown. AG3 and AT9 displayed growth inhibition against*M. luteus*, but without visible evidence of regrowth. The activity of AG3 and AT9 against *M. luteus* and EMRSA was associated with samples from the OSMAC treatments, and the diameters of the clearance zones were measured and are shown in Fig 3. A representative image of AG3 and AT9 activity against EMRSA is provided in Fig. S1. Although neither strain exhibited visible growth inhibition against *C. albicans*, they both produced a blue–purple pigmented substance in the presence of *C. albicans*, suggesting a chemical response. In a repeat of the agar well-diffusion test, antagonistic activity with clearance was observed by both strains against *C. albicans*, albeit with no association to the OSMAC temperature treatments (Table S3).

The agar well-diffusion test, which included an additional centrifugation step to remove cellular material, showed no bacterial regrowth and no antimicrobial activity. On a cross-streak assay (a deferred antagonism assay), the original positive results were replicated in AG3, whereby this isolate exhibited activity against *C. albicans* and *M. luteus*, upon culturing for 7 days at 28°C. This activity was observed in four out of five replicates. The activity was not observed upon culturing AG3 for the same duration at 4°C. Strain AG3 did not display any activity against EMRSA at either temperature. Isolate AT9 did not show any activity in the cross-streak assay upon culturing at both 4°C and 28°C. The patchy distribution of activity could be indicative either of an unstable compound(s) or variable expression of the BGC responsible, therefore the whole genome sequences of the two bacterial strains were further analysed. Results of the antimicrobial tests in which positive results were detected for AG3 and AT9, as well as their replicability are summarized in Table S3.

### Genome sequencing and taxonomic assignment of strains AG3 and AT9

Antarctic invertebrate isolates AG3 and AT9 were identified as belonging to the genera, *Pseudomonas* and *Psychrobacter*, through Sanger sequencing of the 16S rRNA gene region V3–V4. Hybrid sequencing of the two genomes produced a contiguous genome for strain AG3, and an almost complete (3 contigs) genome for AT9 (Table S4). The hybrid draft genome sequence data were then used to obtain more precise species assignments by comparing them with other genome assemblies of closely related type strains in TYGS.

**Figure 4.**
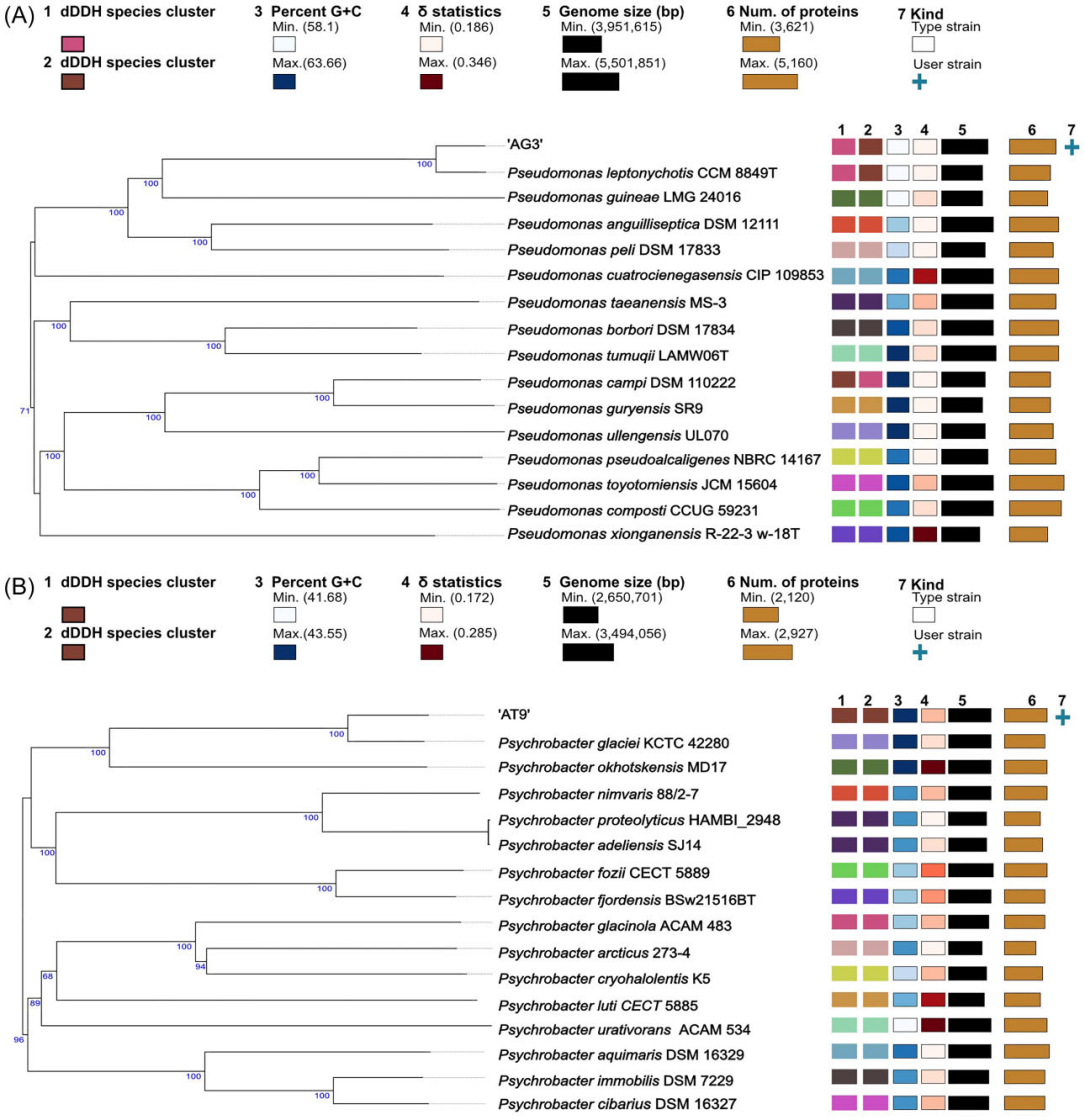
Phylogenetic tree of (A) AG3 and (B) AT9. The tree was produced in TYGS, which uses FASTME 2.1.4 for tree inference from GBDP distances and was calculated from whole genome sequences. The branch lengths are scaled according to GBDP distance formula d5, and the numbers which are shown above the branches indicate GBDP pseudo-bootstrap support values from 100 replications. The coloured boxes within the numbered rows are indicative of the (1) species cluster (2), subspecies cluster (3), percentage G+C content (4), δ values (5), genome size (6), number of proteins (7), and the strain kind. For (1) and (2) species with the same colour belong to the same species and species subcluster, respectively. For (3) and (4) the intensity of the colour corresponds to the percentage G+C content and δ value, i.e. a darker colour represents a higher value. For (5) and (6) the width of the bar corresponds to the genome size (bp) and number of proteins, i.e. a wider bar has a larger genome size and higher number of proteins.

Initially, the ANIb between the two isolates vs. type strains of *Pseudomonas* and *Psychrobacter* was calculated. Strain AG3 was identified as highly similar to *P. leptonychotis* CCM 88498T (isolated from a Weddell seal) (NCBI Taxon ID 2448482), sharing an ANIb of 97.32% (Nováková et al. 2020). This was supported by a dDDH value of 80.1%. However, in comparison to *P. leptonychotis*, the genome of strain AG3 was 379 252 bp longer, with a slightly lower GC content (58.51% compared with 58.80%) (Table 2). A phylogenetic tree based on the 15 closest whole genome sequences to AG3 was constructed on the TYGS. The phylogenetic tree placed AG3 and *P. leptonychotis* in the same species cluster and subspecies cluster and this was strongly supported by a bootstrap of 100. The clade in which strain AG3 and *P. leptonychotis* were placed further included *P. guineae* LMG 24016 (isolated from Antarctic soil) (Bozal et al. 2007) and two marine-associated bacteria, *P. anguilliseptica* DSM 12111 (fish pathogen originally isolated from Japanese eel) (Wiklund and Bylund 1990), and *P. peli* DSM 17833 (isolated from a nitrifying inoculum in Gent, Belgium) (Vanparys et al. 2006) (Fig. 4A). Taking all these data into account, strain AG3 is most likely a strain of *P. leptonychotis*.

**Table 2.** Closest relatives of AG3 and AT9 using JSpecies WS and Genome-to-Genome Distance Calculator 3.0.

Isolate	Assembly size (mbp)	Contigs	N50 (kb)	L50	GC (%)	Closest relative	GC diff. (%)	Protein count diff.
AG3	4.7	1	477.3	1	58.51	*Pseudomonas leptonychotis* CCM 8849	0.29	391
AT9	3.4	3	342.8	1	43.55	*Psychrobacter glaciei* KCTC 42280	0.14	76

The GC diff. refers to the difference in GC content between the isolate and its closest relative. The protein count diff. refers to the difference in the number of protein-encoding genes between the isolate and its closest relative.

In contrast, strain AT9 was most closely related to *Psy. glaciei* KCTC 42280, isolated from an Arctic glacier ice core (NCBI Taxon ID 2448482) (Zeng et al. 2016). However, the ANIb results were borderline at 96.3% and the calculated dDDH value was a little below the species assignment threshold at 69.6%. The genome of AT9 is 117 664 bp longer than that of *Psy. glaciei*, with a slightly higher GC content (43.55% compared with 43.41%) (Table 2). A phylogenetic tree based on the 15 closest whole genomes of strain AT9, constructed on the TYGS, placed it as most closely related to, but not belonging to the same species cluster or subspecies cluster as *Psy. glaciei*. The two strains were, however, in the same clade, which further included *Psy. okhotskensis* MD17 (isolated from the coast of the Okhotsk Sea) (Yumoto et al. 2003) (Fig. 4B). Therefore, AT9 could be a novel *Psychrobacter* species based on phylogenomic data.

### Biosynthetic gene clusters in AG3 and AT9

**Figure 5.**
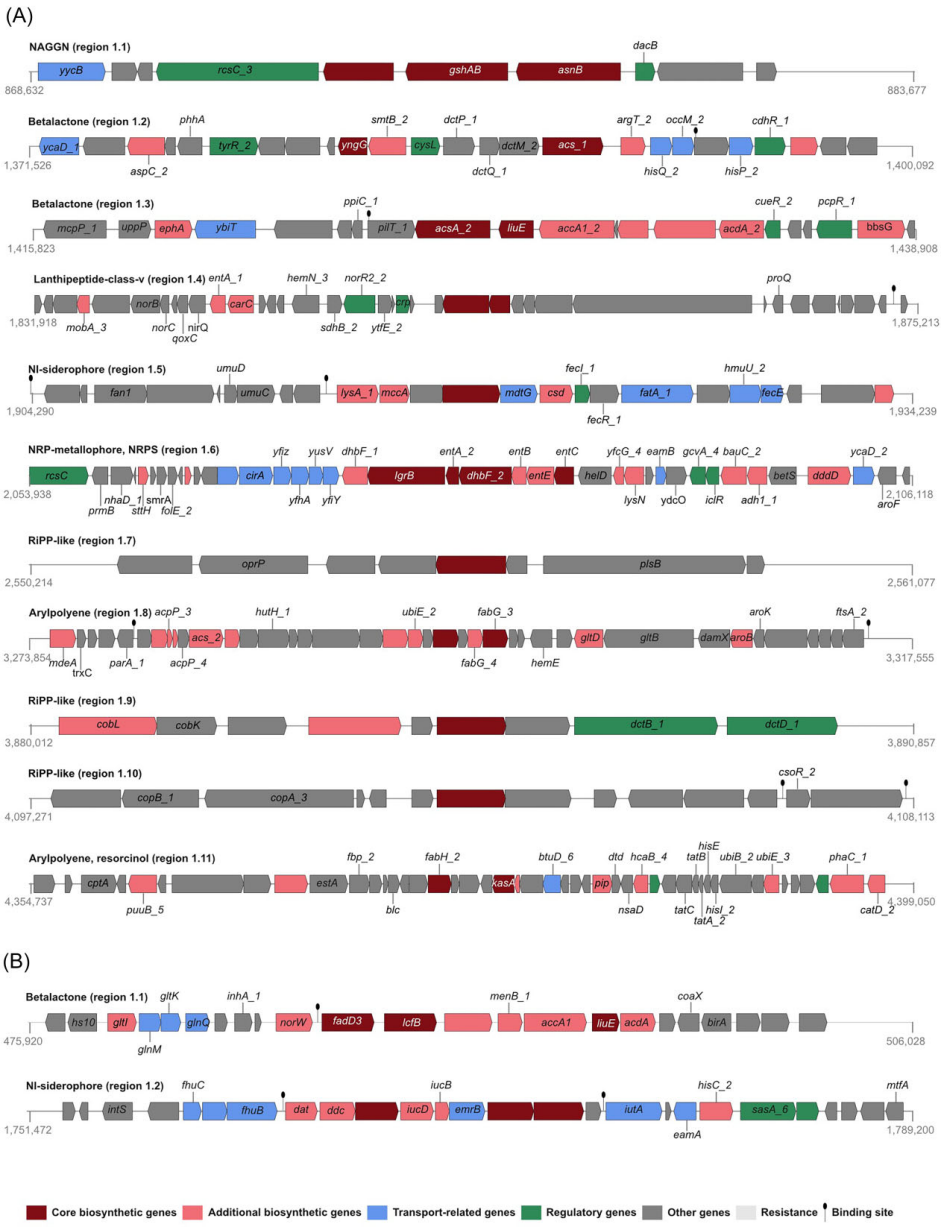
Organization of the BGCs identified in (A) AG3 and (B) AT9 from analysis in AntiSMASH. 7.0. Open reading frames and their direction of transcription are indicated by the arrows. Gene annotations and the nucleotide numbers corresponding to the BGC sequence start and end are provided. Abbreviations: NAGGN, N-acetylglutaminylglutamine amide; NRP, nonribosomal peptide; and RiPP-like, post-translationally modified peptide products.

Potential antimicrobial BGCs in the genomes of strains AG3 and AT9 were identified through antiSMASH. This tool flagged a total of 13 BGCs from the two genomes. The predicted secondary metabolites synthesized by the BGCs were arylpolypenes, betalactones, an N-Acetylglutaminylglutamine amide, nonribosomal peptides (NRPs), a lanthipeptide, post-translationally modified peptide products (RiPP-like), and siderophores (Fig. 5). The predicted BGCs were then matched to the Minimum Information About a Biosynthetic Gene Cluster (MiBIG) database, a repository of BGCs that have been curated and characterized experimentally (Terlouw *et al*. 2023). Out of 13 BGCs, 12 had low similarity scores to the BGCs in the MiBIG database (<0.4), indicating that these BGCs likely produce new or potentially novel compounds (Table S5). Further research is required to characterize and experimentally validate the natural products produced from these BGCs. The Gene Cluster Families (GCFs) most closely related to the BGCs from AG3 and AT9 were identified through BiG-FAM analysis (Table S6). A distance of <900 shows that a BGC has a good match to at least one GCF, while higher distances indicate a poor similarity to existing GCFs and potential novelty (Kautsar et al. 2021). Six BGCs from AG3 were above this threshold. Approximately half (54%) of the matching GCFs have been identified in published genomes of Gram-positive and Gram-negative bacteria, while 31% were exclusive to *Pseudomonas* and *Psychrobacter*.

After the BGCs were identified, ARTS was used to predict the likelihood that the BGCs would produce compounds with antimicrobial properties *in situ*. An active BGC tends to have core genes in its vicinity, multiple copies of itself, and a resistance gene present. ARTS searches for these properties and for domains of unknown functions (DUFs) in the BGC to predict its novelty and thereafter, ranks the BGCs accordingly (Mungan et al. 2020). Based on these criteria, seven BGCs were prioritized (Table 3). All seven BGCs were in the proximity of at least one functional gene, six contained one or more DUFs. Amongst these seven, three were assigned to a BGC group predominantly associated with antimicrobial production. All three were betalactones, of which two (one from AG3 and another from AT9) shared a ∼13% similarity to a BGC that produces a lipopeptide (fengycin) with antagonistic activity against a strain of *C. albicans* (Vanittanakom et al. 1986), as seen in AG3 and AT9 (Table S3). Both betalactone BGCs had positive hits for two resistance encoding genes and the one in AT9 had four essential genes and four DUFs, while the one of strain AG3 had only two essential genes and no DUFs. The potential resistance genes were checked in CARD. Only loose AMR gene hits were associated with the sequences corresponding to the betalactone BGCs. More confident AMR hits (strict) were found across the genomes of AG3 and AT9 (four in AG3 and one in AT9). All five hits were amongst the antibiotic efflux pump gene family with percentage identity matches ranging from 41.35% to 93.44% (Table S7).

**Table 3.** Prioritized BGCs from AG3 and AT9.

Secondary metabolite (most similar known cluster)	Similarity to other clusters	Gene ID	Function	DUF	RESMODEL
**AG3**					
Resorcinol, arylpolyene	91% *Pseudomonas sp*.	TIGR01934	Biosynthesis of cofactors, prosthetic groups, and carriers	5	0
(1% Pf-5 pyoverdine)		TIGR02610	Unclassified		
		TIGR01982	Biosynthesis of cofactors, prosthetic groups, and carriers		
		TIGR00254	Signal transduction		
		TIGR01249	Unclassified		
		TIGR03188	Amino acid biosynthesis		
		TIGR00945	Protein fate		
		TIGR00046	Protein synthesis		
		TIGR00256	Protein synthesis		
		TIGR01411	Protein fate		
		TIGR01410	Protein fate		
Arylpolyene	97% *Pseudomonas peli*	TIGR01464	Biosynthesis of cofactors, prosthetic groups, and carriers	3	0
(45% APE Vf)		TIGR01831	Fatty acid and phospholipid metabolism		
		TIGR01175	Unclassified		
		TIGR01326	Amino acid biosynthesis		
		TIGR02515	Cell envelope		
		TIGR01357	Amino acid biosynthesis		
		TIGR01318	Unclassified		
Betalactone	70% *Pseudomonas xionganensis*	TIGR03718	Unclassified	0	PF00364.17
(13% fengycin)		TIGR00753	Cell envelope		PF01039.17
		TIGR01420	Cellular processes		
Betalactone	75% *Pseudomonas marincola*	TIGR01726	Transport and binding proteins		
		TIGR04381	Regulatory functions	2	0
		TIGR01267	Energy metabolism		
		TIGR01726	Transport and binding proteins		
		TIGR02188	Unclassified		
		TIGR01096	Transport and binding proteins		
NAGGN	100% *Pseudomonas peli*	TIGR00666	Cell envelope		
		TIGR01536	Amino acid biosynthesis	1	0
		TIGR03106	Unclassified		
**AT9**					
Betalactone	95% *Psychrobacter sp*.	TIGR01352	Transport and binding proteins		
(15% plipastatin, 13% fengycin)		TIGR01726	Transport and binding proteins	4	PF00364.17
		TIGR00671	Biosynthesis of cofactors, prosthetic groups, and carriers		PF01039.17
NI-siderophore	96% *Psychrobacter glaciei*	TIGR00709	Central intermediary metabolism	2	0
(27% baumannoferrin A/baumannoferrin B)					

The information on the secondary metabolite, position and similarity to other clusters was taken from analysis in AntiSMASH 7.0 and GeneID and its descriptors were taken from ARTS 2.0. Abbreviations: DUF, domain of unknown function.

## Discussion

This study describes the recovery of 66 bacterial isolates from eight common Antarctic invertebrates: the sea-star, *O. validus*, the brittle star, *O. victoriae*, the sea-cucumbers, *Ec. charcoti; C. georgiana; H. steineni*, the sea anemone *U. antarctica*, the limpet *N. concinna*, and the sea-urchin, *S. neumayeri*. The isolates appear to be closely related to 34 previously reported species (at the 16S rRNA gene level). Using an OSMAC approach, antagonistic antimicrobial activity was demonstrated in two isolates, AG3 (a potential strain of *P. leptonychotis*) and AT9 (a potential novel *Psychrobacter* species). The compound(s) produced appeared to be unstable, or gene cluster expression variable, therefore whole genome sequencing and *in silico* interrogation of BGCs were used to identify potentially novel antimicrobial compounds. Despite demonstrated potential, Antarctic environments are still largely unexplored for natural antimicrobial products and there is a dearth need for increased research efforts in this region (Núñez-Montero and Barrientos 2018, Ramasamy et al. 2023). Only a very small number of the over 17 000 species of marine invertebrates on the Antarctic seabed have been screened for bacteria with antimicrobial properties.

## Diversity of culturable bacteria

The richness of Pseudomonadota in the anemone and mollusc was similar to previous culturing studies on the Antarctic soft coral, *Alcyonium antarcticum, S. neumayeri*, and the Antarctic heart urchin, *Abatus agassizii* (Webster and Bourne 2007, González-Aravena et al. 2016, Schwob et al. 2020). In contrast these previous studies found a greater richness of Bacteroidota and Planctomycetota as compared to Actinomycetota, as seen in some of the echinoderms in this study. Generally, Actinomycetota have not been considered as abundant members of marine communities in culturing studies. However, an amplicon sequencing study identified Actinomycetota as common in the core microbiome of the Antarctic sea-star, *O. validus*, suggesting the differences in richness could be due to isolation methods (Buschi et al. 2023). For instance, freezing invertebrates prior to sampling may have altered cultured bacteria, as Bacteroidota and *Pseudomonas* were only identified in invertebrate hosts that had been freshly sampled. A previous report similarly found a reduction in the detection of Bacteroidota from human stool samples that had been processed after freezing (Bilinski et al. 2022). In addition, since bacteria were selected and sequenced based on morphology, some groups may have been missed, as morphologically similar colonies may not necessarily be identical taxa. Nevertheless, the rich isolation of members of the Actinomycetota and Pseudomonadota families in this study is promising, as these phyla are known to encompass a variety of potent antimicrobial producers (Genilloud 2017, Núñez-Montero et al. 2022).

## OSMAC experiments reveal bacterial thermal and salinity tolerances

All bacteria isolated from the invertebrate host species and subjected to the OSMAC experiments displayed growth at temperatures of at least 22°C and low salinities of 10‰, suggesting all were psychrotolerant and not psychrophilic in nature. These observations are similar to previous data from psychrotolerant bacteria cultured from a range of Antarctic marine environments, including sea-ice and seawater (Helmke and Weyland 1995, Bowman et al. 1997, Hayward et al. 2021). Psychrotolerant bacteria have been found to dominate in seawater, and although the reasons for their thermal resilience are not fully understood, it was hypothesized that psychrotolerant bacteria are better colonizers in seawater, potentially due to the warmer temperatures and at times, increased nutrient deprivation (Bowman et al. 1997, Helmke and Weyland 2004). However, Antarctic bacteria with thermal tolerances exceeding 20°C have been isolated from colder Antarctic environments, including a *Pseudomonas* (strain UTC-1) that was isolated from the Amundsen Scott ice tunnel and was able to survive 34°C despite living at a constant temperature of −50°C (Madigan et al. 2017). These observations are particularly fascinating as the broad thermal ranges of the bacteria contrast the stenothermal nature of their multicellular invertebrate hosts and the stable cold marine environment which they occupy (between –1°C and +2°C and salinities around 33‰) (Clarke et al. 2008, Peck et al. 2014). Nevertheless, there is a possibility that given the very short generation times of bacteria, there could be some adaptation to laboratory conditions, which could potentially explain some of the differences seen between the ecological temperatures that the bacteria naturally live in and the OSMAC conditions tested. This temperature paradox warrants further investigation.

OSMAC approaches successfully led to the production of antimicrobial activity in two isolates (strains AG3 and AT9) (Fig. 3). Temperature can influence antagonistic relationships, for instance, higher temperatures have been observed as stimulants of antagonism in some Arctic soil bacteria strains (Prasad et al. 2011). Furthermore, a temperature increment of 7°C led to a 20% increase in the number of antagonistic interactions between bacteria cultured from the non-polar coral, *Pocillopora damicronis* (Guo et al. 2022). The role of temperature in regulating antagonism could be mediated by its influence on gene regulation of antimicrobial synthesis and the bacterial growth rate (Guo et al. 2022). Indeed, Liao et al. (2009) explored the influence of temperature on regulating the BGC responsible for an antifungal product of *Streptomyces hygroscopicus* 5008, and found optimal culturing temperatures which were associated with an upregulation of transcriptional elements and a key enzyme involved in BGC expression and biosynthesis. Therefore, understanding interactions between temperature and antimicrobial activity can be vital for making decisions on antimicrobial assays. Standard antimicrobial (phenotypic) assays are particularly problematic when screening Antarctic microorganisms because although these bacteria had optimal temperatures ranging from 10°C to 30°C, these are still below the optimal growth temperature of the pathogenic bacteria used in the tests (37°C). These differences in temperature survivability mean that it is either not possible, or difficult, to conduct direct antagonistic experiments between Antarctic bacteria and pathogens. Although studies have found an effect of salinity on antimicrobial production (Ng et al. 2014), the influence of salinity on antimicrobial activity is not apparent from the results of this study, as antimicrobial activity was observed in elevated temperature treatments at a salinity of 10‰ and 22‰. Future studies are required to investigate whether altering salinity provides an additive or suppressive effect on antimicrobial activity. In addition, some isolates were unable to grow well in liquid culture and could not be used for the OSMAC liquid media experiments, highlighting the role of media and culture choice for antimicrobial assessment.

## Antagonistic antimicrobial activity

Extreme conditions in the Antarctic may promote antibiotic-mediated antagonism (Bell et al. 2013, Pantůček et al. 2018). The strains AG3 and AT9 produced antagonistic activity against two Gram-positive bacteria (*M. luteus* and EMRSA) and a fungal pathogen (*C. albicans*). Previous observations suggested phylogenetically distant bacteria are more likely to compete and share antagonistic relationships (Hentschel et al. 2001). Such antagonistic capabilities could confer a competitive advantage against neighbouring bacteria (and fungi) and provide protection to the host. The lack of activity of AG3 and AT9 against the Gram-negative bacteria (*E. coli* and *A. baumannii*) could be suggestive of compound(s) of narrow spectrum that target specific cellular wall components in *M. luteus*/*C. albicans*. Moreover, as the cross-streak assay ensured no physical contact between the pathogenic strains and AG3/AT9, it may suggest that the compound(s) produced by AG3 against *M. luteus* and *C. albicans* in the cross-streak assay were contact-independent, whereas the compound(s) produced by both AT9 and AG3 in the well-diffusion test against EMRSA, may have been contact-dependent. For the repeat agar well-diffusion assay, the isolates had to undergo a freeze-thaw cycle which may have influenced antimicrobial expression and supported the reproducible activity against *C. albicans* or loss of activity against EMRSA and *M. luteus*. Further experimental investigations are required to determine the effect of temperature, freezing, and antimicrobial test (contact vs. non-contact) on the antagonism displayed by AG3 and AT9. These could involve transcriptomic analysis of AG3/AT9 across a range of conditions, overlaid with examination of antagonistic activity in those conditions. Optimal conditions for AG3/AT9 antagonism could then be repeated with inhibitors of well-known antibiotic mechanisms of actions to provide further insights into the compound(s) target or mechanism of action. Nevertheless, the potential instability of the compound(s) produced by strains AG3 and AT9 or their variable BGC expression emphasizes the value of genome mining for revealing bacterial biosynthetic potential and enabling alternative methods of antibiotic discovery.

## Biosynthetic gene cluster potential of strains AG3 and AT9

Unsurprisingly, whole genome taxonomy provided higher species level resolution than the 16S rRNA gene sequence (V3–V4 PCR amplified region) and identified AG3 as a potential strain of the species *P. leptonychotis* (Nováková et al. 2020). Strain AT9 was identified as a potentially novel species based on phylogenomic data, emphasizing the prospect of Antarctic marine environments for the discovery of novel bacterial strains with antimicrobial activity. It is worth mentioning that phylogenomic placements were conducted using TYGS, which offers rapid and user-friendly inference methods, but constructs phylogenetic trees using ‘greedy-with-trimming pseudo bootstrapping for branch support’, and more representative sequences and better-performing algorithms would be required for more robust taxonomic assignments.

A total of 13 BGCs were identified from the genomes of strains AG3 and AT9, many of which had low similarities to experimentally characterized BGC clusters in the MiBIG database and GCFs in BiG-FAM. Previous studies investigating BGCs in cultured Antarctic bacteria and uncultured bacteria have also found low sequence similarities of Antarctic bacteria BGCs to previously experimentally characterized BGCs, proposing this to be an outcome of the extreme environmental conditions, which favour a genetically divergent bacterial community (Brotherton et al. 2018, Núñez-Montero et al. 2022, Waschulin et al. 2022, Medeiros et al. 2024). This is exemplified in this study by the finding that seven BGCs contained unknown domains, which are indicative of novelty.

Three BGCs were assigned as betalactones, a structural group which contains potent antifungal compounds and properties denoting antimicrobial activity (Tomoda et al. 2004, De Pascale et al. 2011). Additionally, two of the betalactone BGCs shared a low similarity (13%) with fengycin, an antifungal product produced by *B. subtilis* (Vanittanakom et al. 1986, Zhou et al. 2023). Fengycin is an unstable lipopeptide synthesized by the NRPs pathway. While this compound has been demonstrated as potentially useful in biocontrol and pharmaceuticals applications, exploitation has been limited due to its complex production, and low yields (Yin et al. 2024). This is interesting considering the instability of the compound(s) produced by strains AG3 and AT9 in this study. However, unlike fengycin, AntiSMASH predicted the BGC produced by AG3 and AT9 as a product of the betalactone pathway rather than the NRPs pathway, which may suggest alternative biosynthesis and potentially novel functionality (Yin et al. 2024). ARTS identified unknown resistance genes associated with two of the betalactones, which could be suggestive of an active BGC (Mungan et al. 2020). The presence of only loose AMR gene hits in the betalactone BGC sequences, upon CARD analysis, may support the novelty suggested by ARTS. Future work would involve enhanced computational analysis of these BGCs to provide increased confidence in novelty prior to experimental examination.

The remaining prioritized BGCs were two arylpolyenes, an Ni-siderophore and an N-Acetylglutaminylglutamine amide, which are groups of BGCs known to harbour diverse molecules that are essential for bacterial protection and can be repurposed for alternative biotechnological applications, such as the production of renewable synthetic dyes (Pailliè-Jiménez et al. 2020, Abdel-Mageed et al. 2021). Such a BGC may be of particular interest, considering the blue–purple substance produced by AG3 and AT9 in the presence of *C. albicans*.

## Conclusion

The extreme Antarctic environment supports a genomically distinct bacterial community with specialized metabolic functionality that can be exploited for antimicrobial discovery. This notion was supported in this study, whereby, two bacterial isolates demonstrated antagonistic activity against two Gram-positive pathogens and a pathogenic fungus, in an OSMAC experiment. Furthermore, *in-silico* genome mining revealed the presence of gene clusters associated with antimicrobial production. It was also shown that the Antarctic marine environment is a rich source of members of the Pseudomonodata and Actinomycetota phyla, as well as psychrotrophic halotolerant bacteria. Altogether, this study highlights the pressing need to heighten antimicrobial discovery efforts in the Antarctic marine environment, while emphasizing the role of OSMAC in amplifying the success of these efforts.

## Supplementary Material

xtaf004_Supplemental_Files

## Data Availability

Cultured bacterial 16S rRNA gene sequences (V3–V4) region were deposited at DDBJ/ENA/GenBank under the accession numbers (PQ101178–PQ101241). Hybrid assemblies and associated raw Illumina and Nanopore sequence data have been deposited at DDBJ/ENA/GenBank and SRA, respectively, and can be found with the following: (1) BioProject, (2) BioSample, and (3) accession numbers [AT9: (1) PRJNA1141887, (2) SAMN42904466, and (3) SRR30237409 (Illumina), SRR30237408 (Nanopore), and JBKOTW000000000 (Hybrid)] [AG3: (1) PRJNA1141919, (2) SAMN42911458, and (3) SRR30035872 (Illumina), SRR30035871 (Nanopore), and CP180477 (Hybrid). The Prokka annotations and analysis outputs from antiSMASH and CARD have been submitted to Zenodo and can be accessed through the following DOI: https://doi.org/10.5281/zenodo.15118396.
