# Supplementary material for: Psychrotrophic Antarctic marine bacteria as potential reservoirs for novel antimicrobial genes
Source: FEMS Microbes. 2025 Apr 15;6:xtaf004. doi: 10.1093/femsmc/xtaf004 (PMC12032627; doi:10.1093/femsmc/xtaf004)
Supplement: xtaf004_Supplemental_Files [file xtaf004_supplemental_files.zip › FEMSMC-2024-042.R2 one sentence summary.docx]

Bacteria taken from Antarctic marine creatures live in a perpetually cold environment but can grow at moderate temperatures and produce novel chemicals for use against human disease.
