# Supplementary material for: Psychrotrophic Antarctic marine bacteria as potential reservoirs for novel antimicrobial genes
Source: FEMS Microbes. 2025 Apr 15;6:xtaf004. doi: 10.1093/femsmc/xtaf004 (PMC12032627; doi:10.1093/femsmc/xtaf004)
Supplement: xtaf004_Supplemental_Files [file xtaf004_supplemental_files.zip › Supplementary Data_References.docx]

**Full length references for Supplementary Data, Table S1**

Al Khudary R, Stösser NI, Qoura F *et al*. *Pseudoalteromonas arctica* sp. nov., an aerobic, psychrotolerant, marine bacterium isolated from Spitzbergen. *Int J Syst Evol Microbiol* 2008, DOI: 10.1099/ijs.0.64963-0.

An S-Y & Yokota A. The status of the species *Leifsonia rubra* Reddy et al. 2003. Request for an Opinion. *Int J Syst Evol Microbiol* 2007, DOI: https://doi.org/10.1099/ijs.0.64954-0.

Bakermans C, Ayala-del-Río HL, Ponder MA *et al*. *Psychrobacter cryohalolentis* sp. nov. and *Psychrobacter arcticus* sp. nov., isolated from Siberian permafrost. *Int J Syst Evol Microbiol* 2006, DOI: https://doi.org/10.1099/ijs.0.64043-0.

Bowman JP, Nichols CM & Gibson JAE. *Algoriphagus ratkowskyi* gen. nov., sp. nov., *Brumimicrobium glaciale* gen. nov., sp. nov., *Cryomorpha ignava* gen. nov., sp. nov. and *Crocinitomix catalasitica* gen. nov., sp. nov., novel flavobacteria isolated from various polar habitats. *Int J Syst Evol Microbiol* 2003, https://doi.org/10.1099/ijs.0.02553-0.

Bozal N, Montes MJ & Mercadé E. *Pseudomonas guineae* sp. nov., a novel psychrotolerant bacterium from an Antarctic environment. *Int J Syst Evol Microbiol* 2007, DOI: 10.1099/ijs.0.65141-0.

Bozal N, Montes MJ, Miñana-Galbis D *et al*. *Shewanella vesiculosa* sp. nov., a psychrotolerant bacterium isolated from an Antarctic coastal area. *Int J Syst Evol Microbiol* 2009, DOI: https://doi.org/10.1099/ijs.0.000737-0.

Busse HJ. Review of the taxonomy of the genus Arthrobacter, emendation of the genus Arthrobacter sensu lato, proposal to reclassify selected species of the genus Arthrobacter in the novel genera Glutamicibacter gen. nov., Paeniglutamicibacter gen. nov., Pseudoglutamicibacter gen. nov., Paenarthrobacter gen. nov. and Pseudarthrobacter gen. nov., and emended description of *Arthrobacter roseus*. *Int J Syst Evol Microbiol* 2016, DOI: https://doi.org/10.1099/ijsem.0.000702.

Chaudhary DK & Kim J. *Sphingomonas olei* sp. nov., with the ability to degrade aliphatic hydrocarbons, isolated from oil-contaminated soil. *Int J Syst Evol Microbiol* 2017, DOI: 10.1099/ijsem.0.002010.

Gallego V, Sánchez-Porro C, García MT *et al*. *Massilia aurea* sp. nov., isolated from drinking water. *Int J Syst Evol Microbiol* 2006, DOI: https://doi.org/10.1099/ijs.0.64389-0.

Ganzert L, Bajerski F, Mangelsdorf K *et al*. *Arthrobacter livingstonensis* sp. nov. and *Arthrobacter cryotolerans* sp. nov., salt-tolerant and psychrotolerant species from Antarctic soil. *Int J Syst Evol Microbiol* 2011, DOI: https://doi.org/10.1099/ijs.0.021022-0.

Han JR, Wang K, Zhang J *et al*. *Polaribacter tangerinus* sp. nov., isolated from sediment in a sea cucumber culture pond. *Int J Syst Evol Microbiol* 2017, DOI: 10.1099/ijsem.0.002369.

Han SK, Nedashkovskaya OI, Mikhailov VV *et al*. *Salinibacterium amurskyense* gen. nov., sp. nov., a novel genus of the family Microbacteriaceae from the marine environment. *Int J Syst Evol Microbiol* 2003, DOI: 10.1099/ijs.0.02627-0.

Jung SY, Lee MH, Oh TK *et al*. *Psychrobacter cibarius* sp. nov., isolated from jeotgal, a traditional Korean fermented seafood. *Int J Syst Evol Microbiol* 2005, DOI: https://doi.org/10.1099/ijs.0.63398-0.

Kim KH, Roh SW, Chang HW *et al*. *Pseudomonas sabulinigri* sp. nov., isolated from black beach sand. *Int J Syst Evol Microbiol* 2009, DOI: 10.1099/ijs.0.65866-0.

Lee DW, Lee JM, Seo JP *et al*. *Phycicola gilvus* gen. nov., sp. nov., an actinobacterium isolated from living seaweed. *Int J Syst Evol Microbiol*  2008, DOI: 10.1099/ijs.0.65283-0.

Li B, Furihata K, Ding LX *et al*. *Rhodococcus kyotonensis* sp. nov., a novel actinomycete isolated from soil. *Int J Syst Evol Microbiol* 2007, DOI: 10.1099/ijs.0.64770-0.

Li WJ, Chen HH, Zhang YQ *et al*. *Nesterenkonia halotolerans* sp. nov. and *Nesterenkonia xinjiangensis* sp. nov., actinobacteria from saline soils in the west of China. *Int J Syst Evol Microbiol* 2004, DOI: https://doi.org/10.1099/ijs.0.02935-0.

Lin P, Yan ZF, Won KH *et al*. *Paracoccus hibiscisoli* sp. nov., isolated from the rhizosphere of Mugunghwa (*Hibiscus syriacus*). *Int J Syst Evol Microbiol* 2017, DOI: 10.1099/ijsem.0.001990.

López-López A, Rogel MA, Ormeño-Orrillo E *et al*. *Phaseolus vulgaris* seed-borne endophytic community with novel bacterial species such as *Rhizobium endophyticum* sp. nov. *Syst Appl Microbiol* 2010, DOI: 10.1016/j.syapm.2010.07.005.

Maruyama A, Honda D, Yamamoto H *et al*. Phylogenetic analysis of psychrophilic bacteria isolated from the Japan Trench, including a description of the deep-sea species *Psychrobacter pacificensis* sp. nov. *Int J Syst Evol Microbiol* 2000, DOI: https://doi.org/10.1099/00207713-50-2-835.

Montecillo JAV & Bae H. Reclassification of *Brevibacterium frigoritolerans* as *Peribacillus frigoritolerans* comb. nov. based on phylogenomics and multiple molecular synapomorphies. *Int J Syst Evol Microbiol* 2022, DOI: https://doi.org/10.1099/ijsem.0.005389.

Mykytczuk NCS, Wilhelm RC & Whyte LG. *Planococcus halocryophilus* sp. nov., an extreme sub-zero species from high Arctic permafrost. *Int J Syst Evol Microbiol* 2012, DOI: https://doi.org/10.1099/ijs.0.035782-0.

Pathom-aree W, Nogi Y, Sutcliffe IC *et al*. *Williamsia marianensis* sp. nov., a novel actinomycete isolated from the Mariana Trench. *Int J Syst Evol Microbiol* 2006, DOI: https://doi.org/10.1099/ijs.0.64132-0.

Reddy GS, Aggarwal RK, Matsumoto GI *et al*. *Arthrobacter flavus* sp. nov., a psychrophilic bacterium isolated from a pond in McMurdo Dry Valley, Antarctica. *Int J Syst Evol Microbiol* 2000, DOI: 10.1099/00207713-50-4-1553.

Reddy, GSN, Prakash JSS, Srinivas R et al. *Leifsonia rubra* sp. nov. and *Leifsonia aurea* sp. nov., psychrophiles from a pond in Antarctica. *International journal of systematic and evolutionary microbiology* 2003;*53*:977-84.

Romanenko LA, Lysenko AM, Rohde M et al. *Psychrobacter maritimus* sp. nov. and *Psychrobacter arenosus* sp. nov., isolated from coastal sea ice and sediments of the Sea of Japan. *Int J Syst Evol Microbiol* 2004, DOI: https://doi.org/10.1099/ijs.0.63096-0.

Romanenko LA, Tanaka N, Svetashev VI *et al*. *Pseudomonas glareae* sp. nov., a marine sediment-derived bacterium with antagonistic activity. *Arch Microbiol* 2015, DOI: 10.1007/s00203-015-1103-6.

SantaCruz-Calvo L, González-López J & Manzanera M. *Arthrobacter siccitolerans* sp. nov., a highly desiccation-tolerant, xeroprotectant-producing strain isolated from dry soil. *Int J Syst Evol Microbiol* 2013, DOI: https://doi.org/10.1099/ijs.0.052902-0.

See-Too WS, Ee R, Lim YL *et al*. Complete genome of *Arthrobacter alpinus* strain R3.8, bioremediation potential unraveled with genomic analysis. *Stand Genomic Sci* 2017, DOI: 10.1186/s40793-017-0264-0.

Takekuchi M, Fang CX & Yokota A. Taxonomic Study of the Genus *Brachybacterium*: Proposal of *Brachybacterium conglomeratum* sp. nov., nom. rev., *Brachybacterium paraconglomeratum* sp. nov., and *Brachybacterium rhamnosum* sp. nov. *Int J Syst Evol Microbiol* 1995, DOI: https://doi.org/10.1099/00207713-45-1-160.

Táncsics A, Máthé I, Benedek T *et al*. *Rhodococcus sovatensis* sp. nov., an actinomycete isolated from the hypersaline and heliothermal Lake Ursu. *Int J Syst Evol Microbiol* 2017, DOI: 10.1099/ijsem.0.001514.

Wang Y, Su P, Zhang P *et al*. First report of bacterial wilt disease caused by *Pantoea agglomerans* on the ornamental perennial *Oxalis articulata* in China. *Plant Dis* 2022, DOI: 10.1094/pdis-08-22-1883-pdn

Yi H, Yoon HI & Chun J. *Sejongia antarctica* gen. nov., sp. nov. and *Sejongia jeonii* sp. nov., isolated from the Antarctic. *Int J Syst Evol Microbiol* 2005, DOI: https://doi.org/10.1099/ijs.0.63273-0.

Yumoto I, Hirota K, Sogabe Y *et al*. *Psychrobacter okhotskensis* sp. nov., a lipase-producing facultative psychrophile isolated from the coast of the Okhotsk Sea. *Int J Syst Evol Microbiol* 2003, DOI: 10.1099/ijs.0.02686-0.

Zhang DC, Liu HC, Xin YH *et al*. *Salinibacterium xinjiangense* sp. nov., a psychrophilic bacterium isolated from the China No. 1 glacier. *Int J Syst Evol Microbiol* 2008, DOI: https://doi.org/10.1099/ijs.0.65802-0.

Zhang DC, Schumann P, Liu HC *et al*. *Arthrobacter alpinus* sp. nov., a psychrophilic bacterium isolated from alpine soil. *Int J Syst Evol Microbiol* 2010, DOI: 10.1099/ijs.0.017178-0.

Zhang G, Ren H, Chen X et al. *Sporosarcina siberiensis* sp. nov., isolated from the East Siberian Sea. *Antonie Van Leeuwenhoek* 2014, DOI:10.1007/s10482-014-0217-1.

**Full length references for Supplementary Data, Table S2**

Al Khudary R, Stösser NI, Qoura F *et al*. *Pseudoalteromonas arctica* sp. nov., an aerobic, psychrotolerant, marine bacterium isolated from Spitzbergen. *Int J Syst Evol Microbiol* 2008, DOI: 10.1099/ijs.0.64963-0.

Authman S, Zaid R & Al-Ezee A. Temperature effects on growth of the biocontrol agent *Pantoea agglomerans* (An oval isolate from Iraqi soils). *J Adv Lab Res Biol* 2018;8:2017-85

Bakermans C, Ayala-del-Río HL, Ponder MA *et al*. *Psychrobacter cryohalolentis* sp. nov. and *Psychrobacter arcticus* sp. nov., isolated from Siberian permafrost. *Int J Syst Evol Microbiol* 2006, DOI: https://doi.org/10.1099/ijs.0.64043-0.

Bowman JP, Nichols CM & Gibson JAE. *Algoriphagus ratkowskyi* gen. nov., sp. nov., *Brumimicrobium glaciale* gen. nov., sp. nov., *Cryomorpha ignava* gen. nov., sp. nov. and *Crocinitomix catalasitica* gen. nov., sp. nov., novel flavobacteria isolated from various polar habitats. *Int J Syst Evol Microbiol* 2003, https://doi.org/10.1099/ijs.0.02553-0.

Bozal N, Montes MJ & Mercadé E. *Pseudomonas guineae* sp. nov., a novel psychrotolerant bacterium from an Antarctic environment. *Int J Syst Evol Microbiol* 2007, DOI: 10.1099/ijs.0.65141-0.

Bozal N, Montes MJ, Miñana-Galbis D *et al*. *Shewanella vesiculosa* sp. nov., a psychrotolerant bacterium isolated from an Antarctic coastal area. *Int J Syst Evol Microbiol* 2009, DOI: https://doi.org/10.1099/ijs.0.000737-0.

Chaudhary DK & Kim J. *Sphingomonas olei* sp. nov., with the ability to degrade aliphatic hydrocarbons, isolated from oil-contaminated soil. *Int J Syst Evol Microbiol* 2017, DOI: 10.1099/ijsem.0.002010.

Costa E, Usall J, Teixidó N *et al*. Water activity, temperature, and pH effects on growth of the biocontrol agent *Pantoea agglomerans* CPA-2. *Can J Microbiol* 2002, DOI: 10.1139/w03-001.

Gallego V, Sánchez-Porro C, García MT *et al*. *Massilia aurea* sp. nov., isolated from drinking water. *Int J Syst Evol Microbiol* 2006, DOI: https://doi.org/10.1099/ijs.0.64389-0.

Ganzert L, Bajerski F, Mangelsdorf K *et al*. *Arthrobacter livingstonensis* sp. nov. and *Arthrobacter cryotolerans* sp. nov., salt-tolerant and psychrotolerant species from Antarctic soil. *Int J Syst Evol Microbiol* 2011, DOI: https://doi.org/10.1099/ijs.0.021022-0.

Han JR, Wang K, Zhang J *et al*. *Polaribacter tangerinus* sp. nov., isolated from sediment in a sea cucumber culture pond. *Int J Syst Evol Microbiol* 2017, DOI: 10.1099/ijsem.0.002369.

Han SK, Nedashkovskaya OI, Mikhailov VV *et al*. *Salinibacterium amurskyense* gen. nov., sp. nov., a novel genus of the family Microbacteriaceae from the marine environment. *Int J Syst Evol Microbiol* 2003, DOI: 10.1099/ijs.0.02627-0.

Jung SY, Lee MH, Oh TK *et al*. *Psychrobacter cibarius* sp. nov., isolated from jeotgal, a traditional Korean fermented seafood. *Int J Syst Evol Microbiol* 2005, DOI: https://doi.org/10.1099/ijs.0.63398-0.

Kim KH, Roh SW, Chang HW *et al*. *Pseudomonas sabulinigri* sp. nov., isolated from black beach sand. *Int J Syst Evol Microbiol* 2009, DOI: 10.1099/ijs.0.65866-0.

Lee DW, Lee JM, Seo JP *et al*. *Phycicola gilvus* gen. nov., sp. nov., an actinobacterium isolated from living seaweed. *Int J Syst Evol Microbiol*  2008, DOI: 10.1099/ijs.0.65283-0.

Li WJ, Chen HH, Zhang YQ *et al*. *Nesterenkonia halotolerans* sp. nov. and *Nesterenkonia xinjiangensis* sp. nov., actinobacteria from saline soils in the west of China. *Int J Syst Evol Microbiol* 2004, DOI: https://doi.org/10.1099/ijs.0.02935-0.

Lin P, Yan ZF, Won KH *et al*. *Paracoccus hibiscisoli* sp. nov., isolated from the rhizosphere of Mugunghwa (*Hibiscus syriacus*). *Int J Syst Evol Microbiol* 2017, DOI: 10.1099/ijsem.0.001990.

Liu GH, Liu B, Wang JP *et al*. Reclassification of *Brevibacterium frigoritolerans* DSM 8801(T) as *Bacillus frigoritolerans* comb. nov. Based on Genome Analysis. *Curr Microbiol* 2020, DOI: 10.1007/s00284-020-01964-x.

López-López A, Rogel MA, Ormeño-Orrillo E *et al*. *Phaseolus vulgaris* seed-borne endophytic community with novel bacterial species such as *Rhizobium endophyticum* sp. nov. *Syst Appl Microbiol* 2010, DOI: 10.1016/j.syapm.2010.07.005.

Maruyama A, Honda D, Yamamoto H *et al*. Phylogenetic analysis of psychrophilic bacteria isolated from the Japan Trench, including a description of the deep-sea species *Psychrobacter pacificensis* sp. nov. *Int J Syst Evol Microbiol* 2000, DOI: https://doi.org/10.1099/00207713-50-2-835.

Mykytczuk NCS, Wilhelm RC & Whyte LG. *Planococcus halocryophilus* sp. nov., an extreme sub-zero species from high Arctic permafrost. *Int J Syst Evol Microbiol* 2012, DOI: https://doi.org/10.1099/ijs.0.035782-0.

Pathom-aree W, Nogi Y, Sutcliffe IC *et al*. *Williamsia marianensis* sp. nov., a novel actinomycete isolated from the Mariana Trench. *Int J Syst Evol Microbiol* 2006, DOI: https://doi.org/10.1099/ijs.0.64132-0.

Reddy GS, Aggarwal RK, Matsumoto GI *et al*. *Arthrobacter flavus* sp. nov., a psychrophilic bacterium isolated from a pond in McMurdo Dry Valley, Antarctica. *Int J Syst Evol Microbiol* 2000, DOI: 10.1099/00207713-50-4-1553.

Reddy, GSN, Prakash JSS, Srinivas R et al. *Leifsonia rubra* sp. nov. and *Leifsonia aurea* sp. nov., psychrophiles from a pond in Antarctica. *International journal of systematic and evolutionary microbiology* 2003;*53*:977-84.

Romanenko LA, Lysenko AM, Rohde M et al. *Psychrobacter maritimus* sp. nov. and *Psychrobacter arenosus* sp. nov., isolated from coastal sea ice and sediments of the Sea of Japan. *Int J Syst Evol Microbiol* 2004, DOI: https://doi.org/10.1099/ijs.0.63096-0.

Romanenko LA, Tanaka N, Svetashev VI *et al*. *Pseudomonas glareae* sp. nov., a marine sediment-derived bacterium with antagonistic activity. *Arch Microbiol* 2015, DOI: 10.1007/s00203-015-1103-6.

SantaCruz-Calvo L, González-López J & Manzanera M. *Arthrobacter siccitolerans* sp. nov., a highly desiccation-tolerant, xeroprotectant-producing strain isolated from dry soil. *Int J Syst Evol Microbiol* 2013, DOI: https://doi.org/10.1099/ijs.0.052902-0.

Takekuchi M, Fang CX & Yokota A. Taxonomic Study of the Genus *Brachybacterium*: Proposal of *Brachybacterium conglomeratum* sp. nov., nom. rev., *Brachybacterium paraconglomeratum* sp. nov., and *Brachybacterium rhamnosum* sp. nov. *Int J Syst Evol Microbiol* 1995, DOI: https://doi.org/10.1099/00207713-45-1-160.

Táncsics A, Máthé I, Benedek T *et al*. *Rhodococcus sovatensis* sp. nov., an actinomycete isolated from the hypersaline and heliothermal Lake Ursu. *Int J Syst Evol Microbiol* 2017, DOI: 10.1099/ijsem.0.001514.

Yi H, Yoon HI & Chun J. *Sejongia antarctica* gen. nov., sp. nov. and *Sejongia jeonii* sp. nov., isolated from the Antarctic. *Int J Syst Evol Microbiol* 2005, DOI: https://doi.org/10.1099/ijs.0.63273-0.

Yumoto I, Hirota K, Sogabe Y *et al*. *Psychrobacter okhotskensis* sp. nov., a lipase-producing facultative psychrophile isolated from the coast of the Okhotsk Sea. *Int J Syst Evol Microbiol* 2003, DOI: 10.1099/ijs.0.02686-0.

Zhang DC, Liu HC, Xin YH *et al*. *Salinibacterium xinjiangense* sp. nov., a psychrophilic bacterium isolated from the China No. 1 glacier. *Int J Syst Evol Microbiol* 2008, DOI: https://doi.org/10.1099/ijs.0.65802-0.

Zhang DC, Schumann P, Liu HC *et al*. *Arthrobacter alpinus* sp. nov., a psychrophilic bacterium isolated from alpine soil. *Int J Syst Evol Microbiol* 2010, DOI: 10.1099/ijs.0.017178-0.

Zhang G, Ren H, Chen X et al. *Sporosarcina siberiensis* sp. nov., isolated from the East Siberian Sea. *Antonie Van Leeuwenhoek* 2014, DOI:10.1007/s10482-014-0217-1.
