# Supplementary material for: Psychrotrophic Antarctic marine bacteria as potential reservoirs for novel antimicrobial genes
Source: FEMS Microbes. 2025 Apr 15;6:xtaf004. doi: 10.1093/femsmc/xtaf004 (PMC12032627; doi:10.1093/femsmc/xtaf004)
Supplement: xtaf004_Supplemental_Files [file xtaf004_supplemental_files.zip › Supplementary Data_Table_Legends.docx]

**Table S1. 16S rRNA identification and metadata of bacteria isolated from eight Antarctic marine invertebrates.** The isolate code, the animal and tissue the isolate was taken from, the temperature (4 ˚C or 15 ˚C), the sample type from initial culturing (fresh or frozen), and sampling date are provided. The 16S rRNA V3-V4 gene region of each bacterium isolated was sequenced and searched on the NCBI core nucleotide database using the BLASTn algorithm to find the closest matching relative. Details of the BLASTn search results (percentage identity, e-value, and query cover) are provided. The environment where the closest relatives have been isolated from is also provided alongside a citation. The two isolates (AG3 and AT9) that produced antimicrobial activity were taken for whole genome sequencing and are shown in bold and underlined. Full length references provided in Supplementary Data, References.

**Table S2. Temperature tolerances of closest relatives of cultured bacteria, according to 16S rRNA V3-V4 region.** The thermal limits of the cultured strains corresponding to 34 bacterial species were searched in the literature and used to designate OSMAC temperature culture conditions (Group 1: 22 °C, Group 2: 28 °C, Group 3; 35 °C). 20 out of the 34 bacterial strains could be successfully maintained in culture, and these were used for the OSMAC experiments and are indicated using an asterix (*). Full length references provided in Supplementary Data, References.

**Table S3. Antimicrobial tests results of AG3 and AT9 to indicate replicability.** The results from the antimicrobial tests in which positive results were observed are listed. The culture conditions of AG3 and AT9 (OSMAC treatments or growth at 28 °C or 4 °C for the cross-streak assay), and replicate details are provided. Signs of inhibition are denoted by a positive red sign (+), whilst negative results are denoted by a negative sign (-). The cases with an asterix (*) indicate that AG3/AT9 produced a visible colour change in the test, but no visible signs of clearance.

**Table S4. Genome assembly statistics of AG3 and AT9.**

**Table S5. MiBIG comparison of BGCs from AT9 and AG3 with experimentally characterised BGCs.**

**Table S6. BIG-FAM analysis from AG3 and AT9.** The AntiSMASH job IDs of AG3 and AT9 were run in BiG-FAM. The distances of the BGCs to GCFs in BiG-FAM are provided together with the occurrence of these GCFs in other published genomes.

**Table S7. Prediction of antimicrobial resistance genes in AG3 and AT9 through analysis in CARD.**
