# Supplementary material for: Psychrotrophic Antarctic marine bacteria as potential reservoirs for novel antimicrobial genes
Source: FEMS Microbes. 2025 Apr 15;6:xtaf004. doi: 10.1093/femsmc/xtaf004 (PMC12032627; doi:10.1093/femsmc/xtaf004)
Supplement: xtaf004_Supplemental_Files [file xtaf004_supplemental_files.zip › Supplementary_Data_Figure_Legend.docx]

**Figure S1. Representative image of initial agar well-diffusion assay of AG3 and AT9 against EMRSA.** Areas noted as inhibition are darker in colour and highlighted within the white dashed boxes. All wells shown in the image were inoculated with aliquots of AG3 and AT9 that had been grown in OSMAC conditions. **(AT9)** Areas of bacterial regrowth are visible as light irregular shapes around some of the wells. **(AG3)** Regrowth was observed in areas where inhibition was observed in AG3, however, as colonies of AG3 presented as large opaque smears across the agar surface, the regrowth could not be captured adequately by the camera. Nevertheless, areas of inhibition are easily distinguishable.
