# Supplementary figures and images for: Psychrotrophic Antarctic marine bacteria as potential reservoirs for novel antimicrobial genes
Source: FEMS Microbes. 2025 Apr 15;6:xtaf004. doi: 10.1093/femsmc/xtaf004 (PMC12032627; doi:10.1093/femsmc/xtaf004)

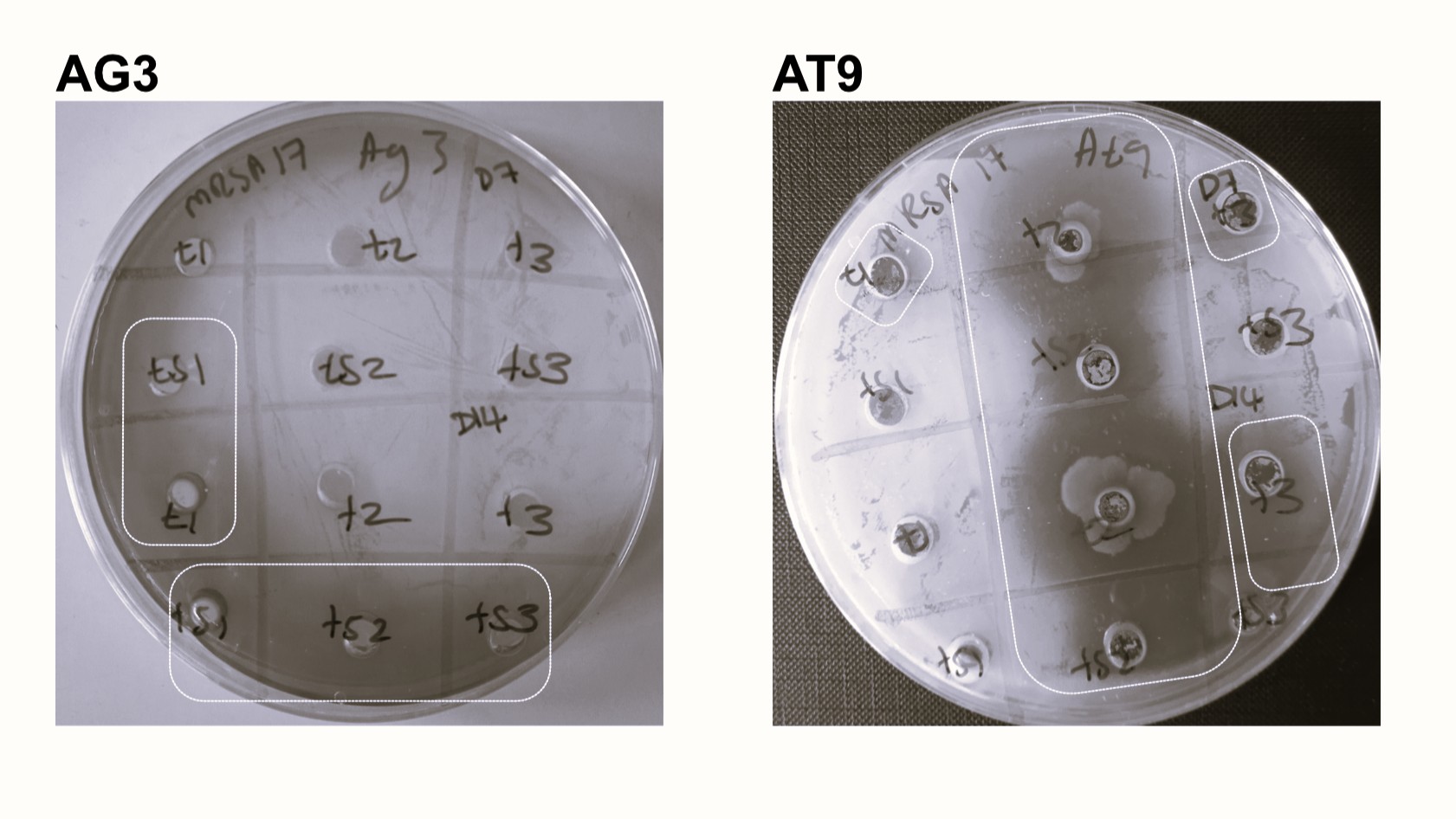

Supplement: xtaf004_Supplemental_Files [file xtaf004_supplemental_files.zip › Figure S1.jpg]
